# Abusive use of Zolpidem as a Result of COVID-19 and Perspectives of Continuity of the Problem in the Post-Pandemic Period

**DOI:** 10.2174/1570159X21666230920123401

**Published:** 2023-09-20

**Authors:** Wesley Dawison de Lima, Michael Douglas da Silva, Eurico de Souza Costa, Francisco Irochima Pinheiro, Eduardo Pereira de Azevedo, Ricardo Ney Cobucci, José Rodolfo Lopes de Paiva Cavalcanti, Fausto Pierdoná Guzen

**Affiliations:** 1 Graduation in Medicine, Health Science Center, State University of Rio Grande do Norte (UERN), Mossoró- RN , Brazil;; 2 Posgraduate Program of Biotechnology, Health School - Potiguar University (UnP), Natal-RN, Brazil;; 3 Department of Tocogynecology, Medical School, Federal University of Rio Grande do Norte (UFRN), Natal-RN, Brazil;; 4 Postgraduate Program in Physiological Sciences, Department of Biomedical Sciences, Faculty of Health Sciences, State University of Rio Grande do Norte (UERN), Mossoró- RN , Brazil;; 5 Postgraduate Program in Health and Society, Department of Biomedical Sciences, Faculty of Health Sciences, State University of Rio Grande do Norte (UERN), Mossoró- RN , Brazil

**Keywords:** Zolpidem, COVID-19, pandemics, substance-related disorders, (GABA-A) receptors, drugs

## Abstract

Zolpidem is a non-benzodiazepine hypnotic drug that works as a positive modulator of Gamma-Amino Butyric Acid-A (GABA-A) receptors, with high selectivity for α1 subunits. Given this selective binding, the drug has a strong hypnotic activity. Social isolation during the SARS-CoV-2 pandemic has contributed to increased rates of anxiety, depression, and insomnia. As a result, studies have pointed to a possible increase in the indiscriminate use of drugs with sedative effects, such as Zolpidem, during the pandemic. The aim of this work was to present prospective evidence that warns of the possibility of the abusive use of Zolpidem even after the pandemic. High rates of addiction to this drug have been reported around the world after the emergence of the coronavirus. Data from the National Survey on Drug Use and Health and from Medicaid support the continuing growth in prescription and indiscriminate use of Zolpidem during the pandemic and afterward. Therefore, there is enough evidence to support the indiscriminate use of this drug since the beginning of the pandemic. Rates of indiscriminate use of sedatives may continue to increase in the post-pandemic period, especially if strict control measures are not taken by health authorities.

## 1 INTRODUCTION

Zolpidem is a psychotropic drug defined as a non-benzodiazepine hypnotic, sedative agent (Z/NBZD), that works as a positive modulator of Gamma-Amino Butyric Acid-A (GABA-A) receptors, found primarily in the brain and spine [1]. In this sense, studies related to the pharmacodynamics of Zolpidem show that it has more affinity for GABA-A receptors in the brain, with high selectivity for α1 subunits [1, 2]. Given this selective binding, some authors claim that this drug has a marked hypnotic effect when compared to its myorelaxant and anticonvulsant activities, which makes it a promising agent for treating insomnia [1, 3].

This hypnotic activity considerably increases the duration of sleep and therefore, mitigates night awakening, which makes Zolpidem a more recommended drug even for older patients when compared to its peers in the benzodiazepine class. This seems to be due to the fact that this drug has very low mortality rates, as they lack the adverse effects of other drugs such as Diazepam and other anxiolytics [1, 4].

Despite being effective in the treatment of insomnia, Zolpidem has the potential to cause reactions and adverse effects that must be considered before use. It affects motor coordination, which increases the risk of falls and the incidence of fractures in the elderly population. Moreover, neuropsychological side effects have been reported, such as a significant reduction in attention and verbal memory, in addition to inducing a state of mental confusion, dizziness and drowsiness during the day [5-8].

Furthermore, studies have reported some cases of decreased awareness of surroundings, greater difficulty in processing thoughts, memory loss, sleepwalking and delirium [9, 10]. Another drug-related side effect described by some authors is the development of depression [11, 12]. The use of Zolpidem along with other central nervous system (CNS) depressant drugs substantially increases the likelihood of overdose [13]. It is imperative to emphasize that Zolpidem is an agent capable of generating euphoric and stimulating effects in its users [12, 14]. In this context, despite being a totally contradictory effect to its mechanism of action, studies have reported that the euphoric and alert effects may result from the use of high doses of this drug [12, 14-17].

As Zolpidem has the potential to cause addiction, a review was carried out on the evidence of growth in prescriptions as well as in the indiscriminate use of this drug after SARS-CoV-2 pandemic.

## 2 LITERATURE REVIEW

The authors searched for articles published in PUBMED, Science Direct, Google Scholar and Cochrane databases using the “mesh” terms COVID-19, Zolpidem, prescriptions, drug abuse and pandemic. The articles that presented data on the growth of abuse and prescription use Zolpidem since the beginning of the pandemic, as decreed by the world health organization, as well the manuscripts that reported cases of Zolpidem misuse, were all included in this search.

Milani *et al*. claim that the social isolation measures that had been taken to mitigate the spread of SARS-CoV-2 have contributed to increased rates of anxiety, depression and insomnia in the overall population. A cohort study conducted by these authors involving United States (US) population, revealed an increase in Z/NBZD and serotonergic drug prescriptions for both men and women along with an increase in benzodiazepine prescriptions for women at the start of the COVID-19 pandemic [18]. The 2020 National Survey on Drug Use and Health (NSDUH) examines the use and misuse of prescription sedatives that include Zolpidem products. Among people aged 12 or older in 2020, 2.2 percent (or 6.2 million people) misused sedatives [19].

A study analyzing the related European Medicines Agency (EMA) EudraVigilance (EV) databases confirmed the diversion potential and the possibility of abuse, misuse, dependence, and withdrawal issues related to all Z-drugs (zaleplon, zopiclone, and zolpidem) [20]. These results, as well as those of the NSDUH, are in line with the growth in rates of mental and sleep disorders observed in Brazil since the beginning of SARS-CoV-2 pandemic. Barros *et al.* concluded that there was an increase of approximately 43.5% in sleep-related problems in the Brazilian population during this period [21].

Saavedra *et al.* stated that a significant increase was clearly observed in the consumption of psychoactive drugs, particularly antidepressants, in the Brazilian capital, which is in accordance with the influence of the pandemic on the mental health of the general population [22]. However, in periods with greater restrictions triggered by the high mortality associated with the coronavirus infection, users of these drugs did not have the support of qualified health professionals to guide the correct and judicious use and therefore, some studies have shown an increase in the indiscriminate use of such agents, including Zolpidem [23, 24].

### 2.1 Abusive use Generating Dependence on Zolpidem

Authors have alerted, especially in the last two decades, about the drug dependence induced by the indiscriminate use of Zolpidem. Despite the fact that this drug was developed as an alternative to others anxiolytics, a series of case reports around the world have cautioned that there is indeed the possibility of development of drug dependence, especially in prolonged use [25, 15]. Orsolini *et al*. reported 8 cases of Zolpidem misuse and concluded that the drug should be prescribed with caution, as it can cause addiction [8]. Chiappini *et al.* also stated that the pandemic had a negative impact on the mental health of the world's population due to fear, social isolation and economic repercussions. Thus, the authors state that prescribers and pharmacists should be warned about possible requests to prescribe more drugs than needed to take home, which might be diverted and abused [26].

In England, high rates of drug addiction have been observed. Davies *et al*. reported that more than 250,000 English people have made indiscriminate use of Z drugs, including Zolpidem, beyond the time recommended by the British health authorities, which is 2 to 4 weeks of use [27]. In Brazil, data collected by the National Controlled Products Management System, which is responsible for overseeing the sales of psychotropics drugs in pharmacies and drugstores, revealed a marked increase in Zolpidem consumption during the pandemic [10, 22]. In Brazil, a substantial growth in search rates for Zolpidem on a drug price comparator platform was observed after the advent of the pandemic when compared to the pre-pandemic period [28]. Analysis of the legal commercialization of this drug has evidenced the emergence of an indiscriminate use of Zolpidem from the beginning of the pandemic until nowadays.

Furthermore, as advocated by Begum *et al.*, prescriptions of Z/NBZD had a constant increase in countries with very high income, such as the United States and Sweden, even in the pre-pandemic period. However, the same authors claimed that restriction measures in Canada, Spain and South Korea, which includes charging for prescriptions as well as health insurance control, might have contributed to a reduction in the number of prescriptions of non-benzodiazepine hypnotics [29].

In Brazil, over the last few years, sales of Zolpidem registered by the National Health Surveillance Agency (ANVISA) increased by 645%, with 10 million boxes sold in 2022 alone [30]. This finding is in accordance with that found by Medcaid, a US public health program focused on serving the less financially favored sections of the population. Accordingly, there was an increase in the prescription of Zolpidem and a decrease in that of Lorazepam [30-34], which corroborates a predilection in the medical class for the former drug.

**Fig. (1):**
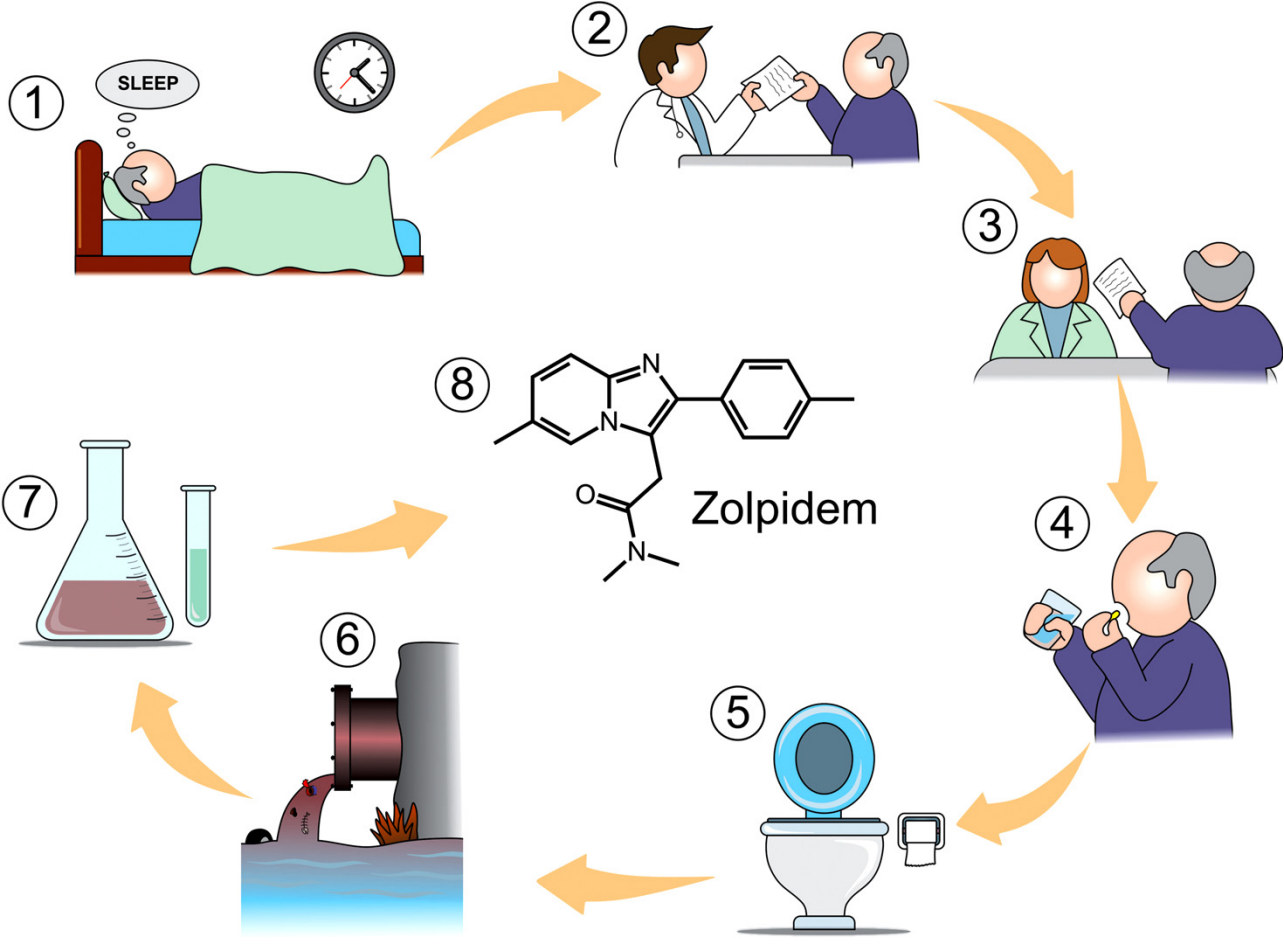
(1) Patient with insomnia (2) Acquisition of a medical prescription (3) Purchase of the medicine at the drugstore (4) Use of the medicine (5) Disposal of the medicine (6) Release into the sewage system (7) Detection in the exception of sewers (United States and Mexico) 8- Zolpidem structure. (Illustration: Francisco Irochima).

Another evidence that supports the indiscriminate use of this drug is the unprecedented finding of the presence of Zolpidem, among other sedatives/hypnotics, in wastewater in the United States and Mexico, as reported by Adhikari *et al*. (Fig. **1**) [35].

The Nantes addict vigilance center, responsible for monitoring the use of Zolpidem in France, proposed two hypothesis that might justify the growing increase in its misuse: one relates to the drug’s hypnotic effects in relation to chronic sleep disorders and the other one relates to younger patients searching for the paradoxical amphetamine-like stimulant effects of zolpidem [15]. The World Health Organization (WHO) and the Food and Drug Administration (FDA) have provided alerts regarding the risks associated with zolpidem, especially drug dependence, thus suggesting dose reduction in some cases [36].

## CONCLUSION

There is enough evidence to support the increased use of Zolpidem since the beginning of the SARS-CoV-2 pandemic. Rates of indiscriminate use of sedatives may continue to increase during the post-pandemic period if strict control measures are not taken by health authorities. Health professionals should be aware of the potential misuse of prescription drugs, especially Zolpidem. In addition, they must recognize the cases of drug abuse and prevent them whenever possible.

If the implementation of regulations that provide efficient control over the distribution and sale of Zolpidem fails, cases involving drug addiction and abusive use of this drug will gradually increase, motivated by factors such as the treatment of insomnia, the search for the sensation of euphoria arising from the consumption of high doses and the reduction in the efficiency of the hypnotic effect caused by long-term use. Therefore, it is concluded that the current indiscriminate use of Zolpidem since the beginning of the SARS-CoV-2 pandemic may become a serious public health problem in the near future. The implementation of successful measures, such as the regulatory framework for the prescription of Zolpidem adopted by France, may contribute to solving this problem, which has already been observed in many countries.

## LIST OF ABBREVIATIONS

GABA-A: Gamma-Amino Butyric Acid-A
CNS: Central Nervous System
EV: EudraVigilance
FDA: Food and Drug Administration
